# Letter of a Valued Correspondent

**Published:** 1871-01

**Authors:** 


### EXTRACT FROM A LETTER OF A VALUED CORRESPONDENT.

"I have just received your note, accompanied by a number of the circulars or notices for the publication of the Georgia Medical Companion. These Circulars I have sent to friends, whom I think, appreciate the importance of such a publication. I am delighted with the movement and its objects, and though not in the practice, I was not surprised by your notice to me of the publication, because you, at least, have had the opportunity to know how much I value the true skill, founded upon the true science of the Physician, who is at the same time a noble, true man. We have some of that stamp in Georgia, but they live under the reproach of being unable or unwilling to unite their talents in the support of a publication like this. They live under the reproach of being mere routinists—mere bookists, by never publishing for the benefit of the Profession, and equally for the benefit of the people, what they see and discover valuable in medical science. They deserve to live under this reproach, as long as they live without a medium of communication with each other from every section of the State—as long as they consent to think and act and learn only for themselves individually. In behalf of the profession I thank you for the effort you are making to wipe out this reproach, and for the opportunity you are giving your professional brethren to aid you in this cause. Is it not a shame that with such a variety of climate and diseases as is furnished by Georgia and the five surrounding Southern States—with such a variety of medicinal properties in the vegetation and the water it affords, there is no organ of the learned profession, to which we all look for light on these vital and lasting questions. Yes, shame, I say—and all success to you in your efforts to wipe it out; and honor to you and your confederates, if you succeed; and you will succeed, if this Journal shall lift up and hold high before the world the standard of medical education—medical honor, personal character, and all the true ethics of the profession. In this you may have a hard battle. If there is as much learning in the profession as ever, it must at the same time be admitted that there is more quackery—more pretention than ever before. It has come to this, that there is more stooping and lowering the character of man and profession for the sake of practice—more unfair ways of getting into practice—more envy, rivalry and personality—more deviations from the true paths of the science—more tendencies to nostrums and specialities than ever before. You must take up arms against all this sea of troubles, and bring back the profession to the standard established by its founders. You must not be afraid to write the truth. The good men of all professions will sustain you. The honor of the profession demands of you to vindicate its rights and character. I am the friend of the Companion, and expect it to make no compromises with error."

The above extract we take from a letter addressed to the senior editor of this Journal, written by a distinguished scholar and high-toned gentleman of Georgia. We make no apology for publishing it, because of the truths it contains, and the importance of their being known. We have taken the only just, and as we think, honorable position, as editors of a medical journal: i. e. a hearty endorsement and cordial support of the cardinal principles of the profession, as embraced in the recognized code of medical ethics. These are simply the utterances and expressions of great, eternal, and invincible moral maxims, founded upon truth. Truth, like everything else emanating from Deity, admits of no compromise. It cannot be sundered, mutilated or suppressed, without deeply wounding its great Author. It admits of no admixture with error. The one was born in Heaven, the other of the—devil. *Veritas, a quocunque dicitur, a Deo est, et vincit omnia.*

